# DNA methylation in the human frontal cortex reveals a putative mechanism for age-by-disease interactions

**DOI:** 10.1038/s41398-019-0372-2

**Published:** 2019-01-29

**Authors:** Brandon C. McKinney, Chien-Wei Lin, Tanbin Rahman, Hyunjung Oh, David A. Lewis, George Tseng, Etienne Sibille

**Affiliations:** 1 Department of Psychiatry, University of Pittsburgh, Pittsburgh, PA USA; 2 Department of Biostatistics, University of Pittsburgh, Pittsburgh, PA USA; 3 Campbell Family Mental Health Research Institute of CAMH, Departments of Psychiatry, and of Pharmacology and Toxicology, University of Toronto, Toronto, ON Canada

## Abstract

A consistent gene set undergoes age-associated expression changes in the human cerebral cortex, and our Age-by-Disease Model posits that these changes contribute to psychiatric diseases by “pushing” the expression of disease-associated genes in disease-promoting directions. DNA methylation (DNAm) is an attractive candidate mechanism for age-associated gene expression changes. We used the Illumina HumanMethylation450 array to characterize genome-wide DNAm in the postmortem orbital frontal cortex from 20 younger (<42 years) and 19 older (>60 years) subjects. DNAm data were integrated with existing normal brain aging expression data and sets of psychiatric disease risk genes to test the hypothesis that age-associated DNAm changes contribute to age-associated gene expression changes and, by extension, susceptibility to psychiatric diseases. We found that age-associated differentially methylated regions (aDMRs) are common, robust, bidirectional, concentrated in CpG island shelves and sea, depleted in CpG islands, and enriched among genes undergoing age-associated expression changes (OR = 2.30, *p* = 1.69 × 10^−27^). We found the aDMRs are enriched among genetic association-based risk genes for schizophrenia, Alzheimer’s disease (AD), and major depressive disorder (MDD) (OR = 2.51, *p* = 0.00015; OR = 2.38, *p* = 0.036; and OR = 3.08, *p* = 0.018, respectively) as well as expression-based MDD-associated genes (OR = 1.48, *p* = 0.00012). Similar patterns of enrichment were found for aDMRs that correlate with local gene expression. These results were replicated in a large publically-available dataset, and confirmed by meta-analysis of the two datasets. Our findings suggest DNAm is a molecular mechanism for age-associated gene expression changes and support a role for DNAm in age-by-disease interactions through preferential targeting of disease-associated genes.

## Introduction

Ameliorating the burden of psychiatric diseases, including schizophrenia (SZ), Alzheimer’s Disease (AD), and major depressive disorder (MDD)^1^ will require advances in their prevention and treatment, and such advances require a neurobiological understanding of their etiology and pathophysiology. Previously, we proposed the Age-by-Disease Model, a neurobiological model for psychiatric diseases^2–4^. This model posits that many psychiatric diseases are, in part, the result of anticipated age-associated changes in the expression of disease-associated genes, and that individual variability in rates of age-dependent changes determines risk or resiliency to develop age-related disorders^2–4^.

The upstream mechanisms giving rise to age-associated gene expression changes are incompletely understood. Candidate mechanisms include loss of telomere integrity, increased oxidative stress, and epigenetic modifications (reviewed in the ref. ^2^). DNA methylation (DNAm), the covalent addition of a methyl group to a cytosine nucleotide in DNA, is an epigenetic modification. Two characteristics of DNAm make it a particularly attractive candidate mechanism. Firstly, changes in DNAm occur throughout the lifespan^5^. DNAm tends to increase with age near genes and decrease with age in intergenic regions^6^. Locus-specific DNAm variability tends to increase with age and is thought to result from each individual’s exposure to a unique set of environmental factors^7–9^. Secondly, changes in DNAm near gene regulatory regions affect local gene expression. Generally, gene expression is negatively correlated with DNAm near the transcriptional start site of a gene, and positively correlated with intragenic DNAm^10^.

In this study, we tested the hypothesis that age-associated DNAm changes contribute to age-associated gene expression changes and, by extension, susceptibility to psychiatric diseases. We predicted that genes that undergo age-associated gene expression changes in the brain would be enriched in differentially methylated regions (DMRs). We also predicted that genes associated with risk for AD, a psychiatric disease associated with advanced age, would be enriched in age-associated DMRs (aDMRs) whereas genes associated with risk for SZ, a psychiatric disease associated with neurodevelopment, would not be enriched in aDMRs. Further, we predicted MDD, a psychiatric disease that can present for the first time or recur at any adult age, would exhibit results intermediate between those for AD and SZ.

## Methods

### Postmortem brains

Brains were recovered during autopsies conducted at the Allegheny County Medical Examiner’s Office, Pittsburgh, PA, following informed consent from the next-of-kin. DSM-IV diagnoses, including substance use disorders, or absence thereof, were determined by clinicians using medical records, structured interviews with surviving relatives, and toxicology reports. The right hemisphere of each brain was cut coronally, immediately frozen, and stored at −80 °C. Samples containing all six cortical layers, but excluding adjacent white matter, were harvested from cryostat sections of the orbital frontal cortex (OFC), specifically Brodmann Areas 11 and 47. Procedures were approved by the University of Pittsburgh Committee for the Oversight of Research and Clinical Trials Involving the Dead and the Institutional Review Board for Biomedical Research.

### Cohort membership

The younger and older groups comprised 22 subjects ≤42 years of age and 22 subjects ≥60 years of age, respectively, without DSM-IV diagnoses or neurologic disease. Age cut-offs were determined by considering availability of subjects with reasonable matching of cofactors, the fact that brain aging is continuous and relatively homogeneous across adult life^11^, and expression levels of biomarkers of brain aging^12,13^. Subjects with the highest (younger group) or lowest (older group) BDNF and SST mRNA levels in our previous study were selected^14^. Because DNAm is highly dependent on race^15–18^ and only 5 subjects were black, they were removed from the cohort, leaving 20 younger, and 19 older, subjects. Groups did not differ with respect to postmortem interval (PMI), RNA integrity number (Agilent, Santa Clara, California, USA), or sex (Table 1 and Supplemental Table 1). Brain pH was slightly higher in the older group but the significance of such a difference is unclear. All data was collected by a researcher blind to age group.

**Table 1:** Group characteristics. Data for continuous variables are presented as group average ± SEM

Group	Younger	Older
Number	20	19
Sex	15 M, 5 F	15 M, 4 F
Race	20 W	19 W
Age (years)*	29.75 ± 2.04	69.32 ± 2.05
PMI (hours)	17.00 ± 1.47	17.58 ± 1.61
Brain pH*	6.65 ± 0.05	6.78 ± 0.05
RIN	8.22 ± 0.14	8.07 ± 0.15
BDNF expression^a*^	24.91 ± 0.68	20.06 ± 0.79
SST expression^a*^	675.37 ± 24.67	385.97 ± 38.34

*F* female, *M* male, *PMI* postmortem interval, *RIN* RNA integrity number, *W* white, *B* black

*Group averages are significantly different (*p* < 0.05)

^a^Microarray signal intensities

### DNA preparation and bisulfite conversion

DNA was isolated from OFC gray matter using AllPrep DNA/RNA/Protein Mini Kit (Qiagen, Valencia, CA, USA) and bisulfite-converted using EZ-96 DNA Methylation Kit (Zymo Research, Irvine, CA, USA), both as per manufacturer’s protocol.

### DNAm arrays

DNAm is the addition of a methyl group to a cytosine nucleotide within the context of a cytosine-phosphate-guanine (CpG) dinucleotide, usually, but also within the context of a cytosine-phosphate-H dinucleotide (CpH; H = adenine, cytosine, or thymine), sometimes^19^. CpGs and CpHs are referred to as “DNAm sites” or “sites” in this manuscript. DNAm was measured at 485,577 sites (482,421 CpG dinucleotides, 3091 CpH dinucleotides, and 65 SNPs) using HumanMethylation450 array (HM450 array; Illumina, San Diego, CA, USA) as per manufacturer’s protocol. The DNAm level at a site was expressed as a *β*-value, the ratio of signal from a methylated probe relative to the sum of both methylated and unmethylated probes.

In an earlier study of somatostatin in the OFC, which included a subset of the same subjects assessed in the present study, a strong correlation was observed between DNAm levels measured by DNAm array and those measured by pyrosequencing^16^.

### Data preprocessing and filtering

Analyses of the data were performed using the R software environment (www.r-project.org).

Data preprocessing (color adjustment, background correction, and quantile normalization) was performed using Bioconductor lumi. Data from poorly hybridizing probes, probes on the X & Y chromosomes, probes with SNPs in the probe or target site, and probes that map to multiple genetic loci were filtered from the dataset. Data from probes corresponding to 317,349 sites remained for analysis^20^ (Supplemental Fig. 1).

### Defining candidate regions and differentially methylated regions

*ß*-values for each subject were smoothed with a 500-base-pair sliding window using Bioconductor methyAnalysis, and then transformed into *M*-values for the purpose of normality approximation. Sites at which DNAm levels differed between groups by two-tailed *t*-test of the *M*-values (*p* < 0.05) were merged into candidate regions (CRs). A CR was defined as a cluster of these sites for which: (a) two consecutive sites were not separated by more than 1000 basepairs and (b) all sites within a given CR were concordant for direction of effect. The rest of sites were treated as isolated CRs. The 317,349 sites were grouped into 267,249 CRs.

The *p*-value for each CR was derived by combining the *p*-values from all sites within the CR using Fisher’s meta-analysis method (FMM). Further, permutation analysis was used. Subject labels were randomly permuted (500 times) and, in each subject permutation, CRs were redefined and *p*-values calculated using FMM. The permuted *p*-value for the ith CR was then calculated as:

$$p_i^ \ast = \frac{{\mathop {\sum}\limits_{b = 1}^{500} {\mathop {\sum}\limits_{j = 1}^{m_b} {I\left( {p_j^{\left( b \right)} \le p_i} \right)} } }}{{\mathop {\sum}\limits_{j = 1}^{500} {m_b} }},$$

where *p*_*i*_ is the meta-analyzed p-value of the observed i-th CR, $p_j^{(b)}$ is the meta-analyzed p-value of the observed j-th CR based on the b-th permutation sample, and m_b_ is the total number of CRs based on b-th permutation sample. False discovery rate (**FDR**) using Benjamini-Hochberg procedure was used to account for multiple testing comparisons. CR-based effect size was meta-analyzed by fixed effect model (FEM) using ß-values. Correlation with expression level for each CR was estimated using the site within the CR that correlated maximally.

A differentially methylated region (DMR) was defined as a CR for which (a) the *q*-value was less than 0.05 and (b) the CR-based effect size was greater than 3%.

### Cell population estimation

Neuron-to-glia proportion in each subject was estimated using a model based on DNAm values from many cell epigenotype specific sites^21^, and it did not differ between groups (Supplemental Figure 4).”

### Gene expression

Gene expression from the OFC of the 39 subjects studied here was previously measured using GeneChip Human Gene 1.1. ST (Affymetrix, Santa Clara, CA, USA), and expression-age associations for each gene were determined by a random intercept model^14,22^. These data were used for enrichment analysis and calculating DNAm-gene expression correlation, and are available for download from Gene Expression Omnibus (GEO; GSE71620).

### Enrichment analysis

Sets of genes that undergo age-associated changes in gene expression^14^ and psychiatric disease risk genes were evaluated for enrichment in age-associated DNAm changes using Fisher’s Exact Tests.

### Pathway and gene ontology analysis

Ingenuity Pathway Analysis (QIAGEN, Redwood City, CA, USA) was used to identify canonical pathways and gene ontologies (molecular function) enriched in genes associated with both age-associated DNAm and expression changes as well as those associated with age-associated DNAm only. *p*-values were ascertained using right-tailed Fisher’s exact tests.

### Replication dataset

Data generated by Jaffe and colleagues^23^ from postmortem dorsolateral prefrontal cortex using HM450K arrays were used for replication analysis. Normalized *β*-values from 133 subjects without DSM-IV diagnoses or neurologic disease, including 102 subjects ≤42 years of age and 31 subjects ≥60 years of age, were downloaded from GEO (GSE74193), and analyzed exactly as described for the primary dataset.

### Meta-analysis of the primary and replication dataset

For each DNAm site, fixed-effect model was used to summarize the effect sizes and *p*-values from the two datasets. The resulting site-based meta-analyzed effect sizes and *p*-values were then used to meta-define CRs following the procedures described for the primary dataset. Region-based effect sizes and correlation were calculated based on meta-defined CRs.

## Results

### Age-associated changes in DNAm are enriched in CpG island shelves and sea, and depleted in CpG islands

**Fig. 1:**
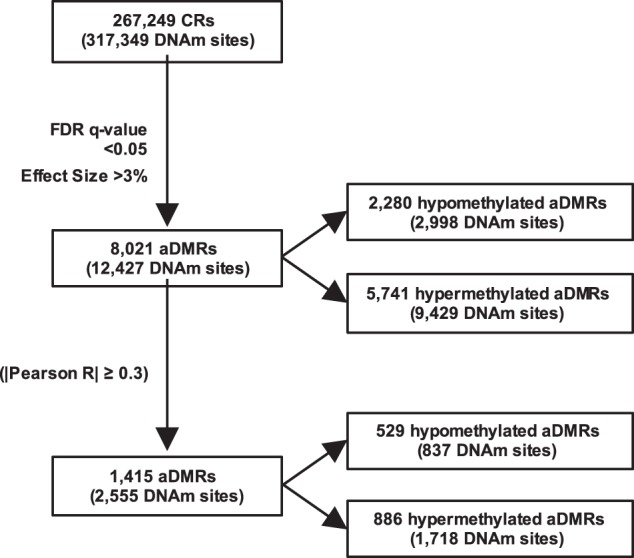
Detecting age-associated differential DNA methylation. The 317,349 DNA methylation (DNAm) sites for which data existed after preprocessing and filtering were grouped into 267,249 candidate regions (CRs). Of the 267,249 CRs, 8021 were differentially methylated between age groups, i.e., differentially methylated regions (aDMRs) (FDR < 0.05 and effect size > 3%). Of the 8021 aDMRs, DNAm at 1415 correlated strongly with local gene expression (|Pearson R| ≥ 0.3)

We classified DNAm sites within age-associated DMRs (aDMR-associated sites; 12,427; Fig. 1) as belonging to one of the following mutually-exclusive genomic regions: *CpG islands* are genomic regions ≥200 basepairs in length with GC content ≥50% and a ratio of observed to expected CpG content ≥0.6^24^; *CpG island shores* are the 2 kilobasepairs flanking outward from CpG islands; *CpG island shelves* are the 2 kilobasepairs flanking outward from CpG island shores; and *CpG island sea* refers to all remaining genomic regions^25^.

**Fig. 2:**
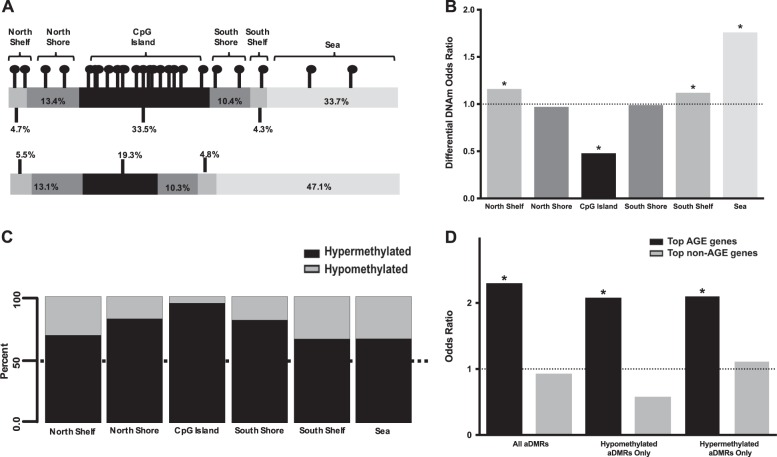
Age-associated changes in DNA methylation are enriched in CpG island shelves and sea, and depleted in CpG islands. **a** Distribution to CpG islands, shores, shelves, and sea of sites that are not differentially methylated between age groups (top), and those that are (bottom). **b** Odds ratio for a differentially methylated site being distributed to each of the genomic locations. **c** Most differentially methylated sites in CpG islands are relatively hypermethylated in the older group. The percentage of sites that are relatively hypermethylated with age in a genomic region decreases with increasing distance from a CpG island. **d** Genes for which expression correlates most strongly with age, i.e., Top-AGE genes, are enriched in aDMRs, both hypomethylated and hypermethylated. Such enrichment is not observed in genes with expression that do not correlate with age, i.e., Top non-AGE genes. *p < 0.05

We found that aDMR-associated sites were not distributed to these genomic regions as expected by chance (*p* = 1.2 × 10^−296^). DNAm sites located in the North and South CpG island shelves (OR = 1.16, *p* = 0.00028 and OR = 1.12, *p* = 0.0094, respectively), and CpG island sea (OR = 1.76, *p* = 2.3 × 10^−201^), are more likely to be associated with an aDMR. In contrast, sites located in CpG islands are less likely (OR = 0.48, *p* = 1.2 × 10^−260^) to be associated with an aDMR (Fig. 2a, b).

### Age-associated hypermethylation is more common in CpG islands

We found that relative hypermethylation in the older group is more common, in general, than relative hypomethylation. Specifically, 71.6% of aDMRs and 75.9% of aDMR-associated sites, are relatively hypermethylated in the older group (Fig. 1). Further, we found that most aDMR-associated sites within CpG islands are relatively hypermethylated in the older group (2276 of 2399 sites, or 94.9%) and that the percentage of relatively hypermethylated aDMR-associated sites becomes smaller as distance from a CpG island increases (Chi-square = 847.6, df = 5, *p* < 2.2 × 10^−16^; Fig. 2c).

### Genes that undergo age-associated changes in expression are enriched in aDMRs

To test if age-associated DNAm changes were preferentially localized to those genes that exhibit age-associated expression changes, we studied those genes in which expression correlated with age in our previous study^14^. The top genes that undergo age-associated changes in gene expression (top AGE genes) were defined as the ~1000 genes with expression that correlated most strongly with age (corresponding to *q*-value ≤ 9.91 × 10^−5^ in BA47 and 9.58 × 10^−5^ in BA11), whereas the top non-AGE genes were defined as the ~1000 genes with expression that correlated most weakly with age (corresponding to *q*-value ≥ 0.43 in BA47 and BA11).

We found that the top AGE genes, but not top non-AGE genes, are enriched in aDMRs (OR = 2.30, *p* = 1.69 × 10^−27^; OR = 0.93, *p* = 0.79; respectively). Top AGE genes are enriched in aDMRs that are relatively hypomethylated (OR = 2.08, *p* = 3.85 × 10^−11^), and relatively hypermethylated (OR = 2.10, *p* = 7.21 × 10^−18^), with age (Fig. 2d, Supplemental Table 2). Notably, the direction of age-associated changes in DNAm and gene expression are inversely related for genes at which age-associated changes in DNA methylation and gene expression converge (Supplemental Fig. 3).

To increase the likelihood of identifying DNAm sites with particular relevance to age-associated gene expression changes, we integrated DNAm and gene expression data. Of the 8021 aDMRs identified, 1415 were found to correlate with local gene expression with a strength of |Pearson’s *R*| ≥ 0.3 (Fig. 1); we henceforth refer to them as expression-correlating aDMRs. Genes that undergo age-associated expression changes are highly enriched in expression-correlating aDMRs (OR = 8.58, *p* = 8.76 × 10^−119^). To gain insight into the potential biological significance of age-associated changes in DNAm, we performed pathway and gene ontology analysis on genes that both undergo age-associated expression changes and have expression-correlating aDMRs annotated to them (*N* = 260; Supplemental Table 3). Further, we performed pathway and gene ontology analysis on only genes to which expression-correlating aDMRs were annotated (*N* = 936) (Supplemental Table 6). Both gene sets were enriched (*p* < 0.05) in distinct canonical pathways and gene ontologies related to neuronal signaling (Supplemental Tables 4, 5, 7 & 8).

### Risk genes for psychiatric diseases are enriched in aDMRs

We sought to determine if risk genes for three psychiatric diseases—SZ, AD, and MDD—were enriched for aDMRs. For SZ, risk genes were defined based on closest proximity to one of the top 108 genome-wide association studies (GWAS)-associated common variants^26^. For AD, 27 genes were defined as risk genes based on closest proximity to one of the GWAS-associated common variants^27,28^, or being one of the genes associated with rare deterministic mutations^29^. For MDD, risk genes were defined based on closest proximity to one of the common variants associated with MDD at *p* < 10^−5^ in a recent large GWAS^30^. DNAm data for 78 SZ, 19 MDD, and 25 AD risk genes were available for analysis (Supplemental Table 9).

**Fig. 3:**
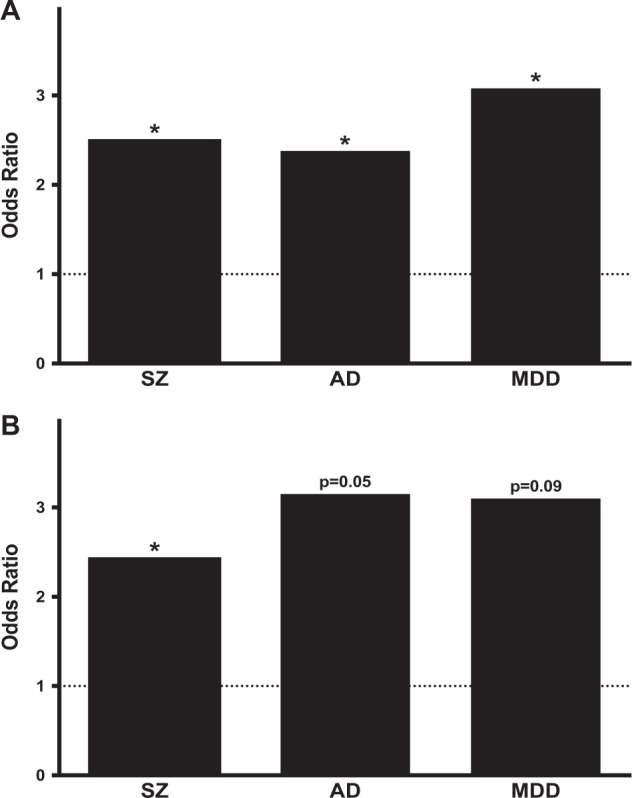
Risk genes for psychiatric disease are enriched in aDMRs. **a** Risk genes for SZ, AD, and MDD are enriched in aDMRs. **b** Risk genes for SZ are enriched in expression-correlating aDMRs. Both AD and aMDD risk genes are enriched in expression-correlating aDMRs with marginal significance but high odds ratio. *p < 0.05

SZ risk genes are enriched in aDMRs (OR = 2.51, *p* = 0.00015) (Fig. 3a) and expression-correlating aDMRs (OR = 2.44, *p* = 0.0013) (Fig. 3b). Of GWAS-associated common variants for SZ, 43 are within a protein-coding gene and DNAm data for 40 were available for analysis (Supplemental Table 10)^26^. Enrichment for aDMRs and expression-correlating aDMRs is more robust (OR = 5.19, *p* = 4.7 × 10^−7^ and OR = 5.53, *p* = 6.3 × 10^−5^; respectively) in this gene subset. AD risk genes are enriched in aDMRs (OR = 2.38, *p* = 0.04) (Fig. 3a) and expression-correlating aDMRs with marginal significance but high odds ratio (OR = 3.15, *p* = 0.05) (Fig. 3b). Similarly, MDD risk genes are enriched in aDMRs (OR = 3.08, *p* = 0.02) (Fig. 3a) and expression-correlating aDMRs with trend-like significance but high odds ratio (OR = 3.10, *p* = 0.09) (Fig. 3b)

### Genes differentially expressed in MDD are enriched in aDMRs

Given the absence of robust genetic associations with MDD, we also assessed for aDMR enrichment among differentially expressed genes in MDD. We previously used meta-analysis to identify 566 genes for which expression consistently differed between MDD and control subjects^31^. DNAm data were available for analysis for 521 of these genes (Supplemental Table 11). These genes were enriched in aDMRs (OR = 1.48, *p* = 0.00012) and expression-correlating aDMRs (OR = 1.91, *p* = 3.5 × 10^−5^) (Supplemental Fig. 2).

### Major findings from our primary dataset are replicated in a independent dataset and confirmed by meta-analysis

We next sought to replicate our findings in an independent dataset (See methods). DNAm sites for which data existed in the replication dataset were grouped into 267,370 CRs, and 4074 were differentially methylated between age groups, i.e., aDMRs. We found that top AGE genes were enriched in aDMRs (OR = 2.19, *p* = 9.24 × 10^−19^). SZ risk genes, including the subset of protein-coding risk genes, were enriched in aDMRs (OR = 2.01, *p* = 0.01 and OR = 3.88, *p* = 0.00017, respectively). AD risk genes were not enriched in aDMRs (OR = 1.37, *p* = 0.37). MDD risk genes and genes differentially expressed in MDD were enriched in aDMRs (OR = 3.32, *p* = 0.02 and OR = 2.01, *p* = 1.3 × 10^−9^, respectively) (Table 2).

**Table 2:** aDMR enrichment of gene sets for aging and psychiatric disease risk in two datasets and meta-analysis

Gene Sets	Primary dataset	Replication dataset	Meta-analysis
*Age*			
All aDMRs	***OR*** ***=*** ***2.30, p*** ***=*** ***1.69*** ***×*** ***10***^*−27*^	***OR*** ***=*** ***2.19, p*** ***=*** ***9.24*** ***×*** ***10***^*−19*^	***OR*** ***=*** ***2.27, p*** ***=*** ***2.52*** ***×*** ***10***^*−23*^
Hypomethylated aDMRs only	***OR*** ***=*** ***2.08, p*** ***=*** ***3.85*** ***×*** ***10***^*−11*^	***OR*** ***=*** ***1.88, p*** ***=*** ***5.55*** ***×*** ***10***^*−7*^	***OR*** ***=*** ***2.28, p*** ***=*** ***2.04*** ***×*** ***10***^*−16*^
Hypermethylated aDMRs only	***OR*** ***=*** ***2.10, p*** ***=*** ***7.21*** ***×*** ***10***^*−18*^	***OR*** ***=*** ***2.21, p*** ***=*** ***2.20*** ***×*** ***10***^*−13*^	***OR*** ***=*** ***2.07, p*** ***=*** ***2.02*** ***×*** ***10***^*−11*^
*SZ*			
SZ risk genes	***OR*** ***=*** ***2.51, p*** ***=*** ***1.50*** ***×*** ***10***^*−4*^	***OR*** ***=*** ***2.01, p*** ***=*** ***0.01***	***OR*** ***=*** ***2.68, p*** ***=*** ***0.00012***
SZ risk genes (protein-encoding subset)	***OR*** ***=*** ***5.19, p*** ***=*** ***4.7*** ***×*** ***10***^*−7*^	***OR*** ***=*** ***3.88, p*** ***=*** ***1.7*** ***×*** ***10***^*−4*^	***OR*** ***=*** ***6.28, p*** ***=*** ***3.6*** ***×*** ***10***^*−8*^
*AD*			
AD risk genes	***OR*** ***=*** ***2.38, p*** ***=*** ***0.04***	OR = **1.37,** ***p*** = 0.37	***OR*** ***=*** ***2.66, p*** ***=*** ***0.0.026***
MDD			
MDD risk genes	***OR*** ***=*** ***3.15, p*** ***=*** ***0.05***	***OR*** ***=*** ***3.32, p*** ***=*** ***0.02***	***OR*** ***=*** ***4.11, p*** ***=*** ***0.004***
Expression-based MDD-associated genes	***OR*** ***=*** ***1.48, p*** ***=*** ***1.2*** ***×*** ***10***^*−4*^	***OR*** ***=*** ***2.01, p*** ***=*** ***1.3*** ***×*** ***10***^*−9*^	***OR*** ***=*** ***1.83, p*** ***=*** ***2.1*** ***×*** ***10***^*−8*^

*DMRs* differentially methylated regions, *OR* odds ratio, *SZ* schizophrenia, *AD* Alzheimer’s disease, *MDD* major depressive disorder

Values in bold italics are statistically significant (*p* < 0.05)

In a meta-analysis of the primary and replication datasets, we found that aDMRs were enriched among top AGE genes (OR = 2.27, *p* = 9.24 × 10^−23^); genetic risk genes for SZ (OR = 2.68, *p* = 0.00012), AD (OR = 2.66, *p* = 0.026), and MDD (OR = 4.11, *p* = 0.0042); as well as genes differentially expressed in MDD (OR = 1.83, *p* = 2.1 × 10^−8^).

## Discussion

In this study, we first characterized DNA methylation (DNAm) differences between younger and older adult subjects in the orbital frontal cortex (OFC). We found that age-associated DNAm changes are common, robust, bidirectional, concentrated in CpG island shelves and sea, and depleted in CpG islands. Next, we tested the hypothesis that age-associated DNAm changes contribute to age-associated gene expression changes and, by extension, susceptibility to psychiatric diseases. Supporting this hypothesis, we found genes that undergo age-associated expression changes as well as genetic risk genes for three psychiatric diseases—SZ, AD, MDD—and genes differentially expressed in MDD are enriched in aDMRs. Further, we replicated the major findings in a large publically-available DNAm dataset, and confirmed them by meta-analysis of the primary and replication datasets. Together, our results provide evidence for DNAm as a mechanism for age-associated gene expression changes in the brain and support a role for DNAm in age-by-disease interactions through preferential targeting of disease-related genes.

### Characterization of age-associated DNAm changes in the human OFC

Our findings with regard to age-associated DNAm changes in the OFC are largely consistent with previous studies of DNAm changes in normal aging human brains using Illumina DNAm arrays^32–36^. We found that the percentage of sites that underwent age-associated changes in DNAm was 3.9% (12,427/317,349), somewhat higher than reported in comparable studies^32–38^. For example, the largest comparable study found that only ~1% of sites assessed exhibited DNAm changes^32^. The fact that all subjects in that study were 66 years of age or older may explain this difference. The rate of DNAm change slows with increasing age^35^, and, paradoxically, brain DNAm signatures may become more similar among individuals after the age of 75^39^. The greater percentage of age-associated DNAm changes in our study may also be explained, in part, by the fact that we selected subjects to maximize group differences in two molecular markers of brain aging (BDNF and SST mRNA levels)^12,13^. Supporting this latter explanation is the observation that only 2.2% (7083/317,349) of sites, are differentially methylated in the replication dataset (from a study in which subjects were not selected using aging biomarkers).

Consistent with the observations of others^40^, we found that age-related DNAm changes were more likely to occur outside of CpG islands, specifically, in CpG island shelves and sea. This observation may reflect the presence of mechanisms that protect CpG islands from de novo methylation^41^. Several studies of age-related DNAm changes using Illumina DNAm arrays report finding a greater percentage of changes occur in CpG islands^32,33,36^. Such reports, while accurate, do not correct for the fact that the probes on Illumina DNAm arrays are biased towards CpG islands.

Our observation that age-associated DNAm hypermethylation was much more common than hypomethylation is consistent with prior reports^6^. This finding, however, likely reflects the fact that the sites probed by Illumina DNAm arrays are biased toward CpG islands, promoters, and genic regions. In one study of peripheral blood that used whole-genome bisulphite sequencing instead of Illumina DNAm arrays, hypermethylation represented only 13% of the age-associated DNAm changes^42^. In fact, most data suggest that the genome becomes globally hypomethylated with age and that the bulk of the DNAm loss occurs at the repetitive sequences in between genes^6^. The pattern of genic hypermethylation and intergenic hypomethylation with age is hinted at in our study by the observation that the likelihood of a site becoming hypermethylated with age appears to be a function of proximity to a CpG island.

### Age-associated changes in gene expression and DNAm

Consistent with our hypothesis, both aDMRs and expression-correlating aDMRs are overrepresented among genes that undergo age-associated changes in expression. These findings suggest that age-associated changes in DNAm may be a mechanism contributing to age-associated expression changes for many genes across the genome. We previously demonstrated that DNAm at sites in a smaller number of genes implicated in psychiatric disease (several BDNF-related and GABA-related genes) correlated strongly with local gene expression^16^.

Pathway and gene ontology analysis of genes at which age-associated expression changes and expression-correlating DMRs converge identified some pathways and gene ontologies previously implicated in brain aging. For example, multiple canonical pathways and gene ontologies related to calcium signaling were identified by these analyses, and calcium signaling is already appreciated as one of the most prominent cellular and molecular functions to be dysregulated in brain aging^43–46^. Indeed, calcium signaling proteins are thought to be promising targets for drugs to treat age-related brain diseases^46^ and a transgenic mouse that overexpresses the L-type calcium channel Ca_v_1.3 in a forebrain-specific manner has been advanced as an animal model of normal brain aging^47^. However, these analyses also identified some pathways and gene ontologies not typically associated with brain aging such as opioid signaling, a potentially promising toper for future investigation high burden of chronic pain in older adults^48–50^. Pathway and gene ontology analysis using only genes to which expression-correlating aDMRs were annotated identified some distinct canonical pathways and gene ontologies from the analyses that also incorporated information about genes that undergo age-associated gene expression. Most notably, this gene set was distinctly enriched in canonical pathways and gene ontologies associated with brain pathology including gliomas and other malignancies as well as neuropathic pain thus suggesting that expression of these genes may only be altered in specific contexts like particular brain pathologies, etc. However, because Illumina DNAm arrays are biased towards particular regions of the genome, results derived from pathway analysis software created for gene expression arrays should be interpreted with caution^51^.

### Age-associated DNAm changes and psychiatric risk genes

Consistent with our prediction, genes associated with psychiatric diseases through genetic studies were enriched in aDMRs. However, the specific sets of risk genes affected were different than predicted. Contrary to our prediction, SZ risk genes were the psychiatric disease-associated gene set that was most strongly enriched in aDMRs (greater OR and lower *p*-value), whereas aDMR enrichment for AD risk genes were the most weakly enriched (lower OR and higher *p*-value). MDD risk genes defined based on genetic studies as well as expression-based MDD-associated genes were enriched in age-associated aDMRs and thus consistent with our prediction,

Our findings that psychiatric disease risk genes are also enriched in expression-correlating aDMRs suggest that DNAm changes in these genes often alter their expression. Together, these observations support a role for age-associated DNAm changes in age-by-disease interactions. Our Age-by-Disease Model posits that these age-associated changes in gene expression then push biological processes in a direction that promotes a physiological state in the brain that is more susceptible to psychiatric diseases like SZ, MDD, and AD when occurring out of their chronological context, i.e., earlier in life^2–4^. Additional pushes by genetic or environmental factors may then be sufficient to generate brain dysfunction that gives rise to psychiatric diseases.

We predicted that any age-associated DNAm changes relevant to SZ etiology and/or pathophysiology would occur during neurodevelopment, be complete by late adolescence, and not be detectable because our age groups did not span the neurodevelopmental period. Our observation that SZ risk genes are enriched in aDMRs instead suggests that age-associated DNAm changes relevant to SZ may occur during neurodevelopment, but also continue as the brain ages into and through adulthood. Indeed, many important developmental genes are associated with DNAm changes during both early development and adulthood^52,53^ The age-associated DNAm changes in SZ risk genes during adulthood may impact the longitudinal course of SZ and suggests a potential mechanism for some of the changes observed in subjects with SZ across adulthood including cortical gray matter thinning^54,55^ and progressive cognitive decline^56,57^.

We predicted that AD risk genes would be enriched in aDMRs because the age groups studied captured the extremes of the transition from younger to older adult, a period during which one might expect AD-related biological processes to be particularly active. The AD risk gene set was enriched in aDMRs, and the potential importance of this observation to AD etiology is supported by recent studies showing that altered DNAm in AD susceptibility genes is associated with AD neuropathology^58,59^. Notably, these studies found cortex-specific *ANK1* hypermethylation to be one of the alterations most strongly associated with AD neuropathology and we found *ANK1* to contain multiple large relatively hypermethylated aDMRs in this study. The fact that AD risk gene set was the psychiatric disease-associated gene set that was most weakly enriched in aDMRs may be explained by the power lost but studying a limited number of genes (*N* = 25 after data filtering). Also, the fact that the definition of ‘risk gene’ differed among SZ, MDD, and AD may have caused divergence from our predictions. Risk genes for SZ and most of the AD risk genes were defined based on their proximity to GWAS-associated common genetic variants for each disease. The genes associated with the rare deterministic genetic mutations for AD were also considered AD risk genes. Because so few common genetic variants have robustly been associated with MDD, risk genes for MDD were defined based on proximity to common genetic variants found to be associated with MDD at the level of *p* < 10^−5^ in a single large study and thus quite different from the robust associations demonstrated for SZ and AD. The role in disease etiology and pathophysiology of risk genes defined in these various ways are likely different, as is the way they would likely interact with DNAm changes. Because MDD has a bimodal age of onset^60,61^ and can first present or recur at any adult age, we predicted that MDD risk genes would be enriched in aDMRs to a degree intermediate between SZ and AD given that our age groups may not be particularly well-suited for detecting age-associated DNAm changes relevant to MDD that is limited to early or late adulthood. Our observation that MDD risk genes, and genes differentially expressed in the brains of MDD subjects, are enriched in aDMRs is consistent with this prediction. The accumulation of age-associated DNAm changes over time in genes associated with MDD may help explain the high rates of depressive symptoms among individuals in late life^62,63^, as we have previously hypothesized^2,3^.

## Conclusion

This study provides further evidence for DNAm as a mechanism contributing to age-associated gene expression changes. Additionally, it provides further support for age-associated DNAm changes as a mechanism for mediating the age-by-disease changes in gene expression previously hypothesized to contribute to the etiology and pathophysiology of psychiatric diseases.

## Supplementary information

Supplemental Text and Figures Merged

Supplemental Table 1

Supplemental Table 2

Supplemental Table 3

Supplemental Table 4

Supplemental Table 5

Supplemental Table 6

Supplemental Table 7

Supplemental Table 8

Supplemental Table 9

Supplemental Table 10

Supplemental Table 11

Supplemental Table 12

Supplemental Table 13
