# Supplementary material for: DNA methylation in the human frontal cortex reveals a putative mechanism for age-by-disease interactions
Source: Transl Psychiatry. 2019 Jan 29;9:39. doi: 10.1038/s41398-019-0372-2 (PMC6351569; doi:10.1038/s41398-019-0372-2)
Supplement: Supplementary file 1 — Supplemental Text and Figures Merged [file 41398_2019_372_MOESM1_ESM.docx]

### SUPPLEMENTARY INFORMATION FOR:

**DNA METHYLATION IN THE HUMAN FRONTAL CORTEX REVEALS A PUTATIVE MECHANISM FOR AGE- BY-DISEASE INTERACTIONS**

Brandon C. McKinney*, Chien-Wei Lin, Hyunjung Oh, David A. Lewis, George Tseng, Etienne Sibille*

### Table of Contents

**Supplementary Figures and Tables**

**Supplemental Figure 1.** Subjects segregated by age group on MDS plot after probe filtering.

**Supplemental Figure 2.** Expression-based MDD-associated genes are enriched in aDMRs and expression- correlating aDMRs

**Supplemental Figure 3.** Direction of age-associated changes in DNA methylation and expression are inversely related for genes at which age-associated changes in DNA methylation and gene expression converge

**Supplemental Figure 4.** Neuron-to-glia proportion does not differ between younger and older age groups.

**Supplemental Table 1.** Characteristics of human subjects. *Submitted as xlsx.*

**Supplemental Table 2.** Genes that undergo age-associated changes in gene expression are enriched in DMRs. *Submitted as xlsx.*

**Supplemental Table 3.** Genes that undergo age-associated changes in gene expression and to which an expression-correlating aDMR is annotated (N=260). *Submitted as xlsx.*

**Supplemental Table 4.** The top 10 canonical pathways identified using the list of genes at which age- associated changes in DNAm and gene expression converged. *Submitted as xlsx.*

**Supplemental Table 5.** The top 10 enriched gene ontology (GO; molecular function) groups identified using the list of genes at which age-associated changes in DNAm and gene expression converged.

*Submitted as xlsx.*

**Supplemental Table 6.** Genes to which an aDMR is annotated but do not undergo age-associated changes in gene expression and (N=936). *Submitted as xlsx.*

**Supplemental Table 7.** The top 10 canonical pathways identified using the list of genes to which an aDMR is annotated but do not undergo age-associated changes in gene expression. *Submitted as xlsx.*

**Supplemental Table 8.** The top 10 enriched gene ontology (GO; molecular function) groups identified using the list of genes to which an aDMR is annotated but do not undergo age-associated changes in gene expression. *Submitted as xlsx.*

**Supplemental Table 9.** Lists of SZ, AD, and MDD risk genes used for enrichment analysis. *Submitted as xlsx.*

**Supplemental Table 10.** Protein-encoding genes that intersect a SZ-associated common genetic variant.

*Submitted as xlsx.*

**Supplemental Table 11.** Expression-based MDD-associated genes. *Submitted as xlsx.*

**Supplemental Table 12.** Complete table of DMRs from primary dataset with annotations and statistics.

*Submitted as xlsx.*

**Supplemental Table 13.** Complete table of DMRs from meta-analysis of primary and replication datasets with annotations and statistics. *Submitted as xlsx.*

# Supplemental Figure 1

###
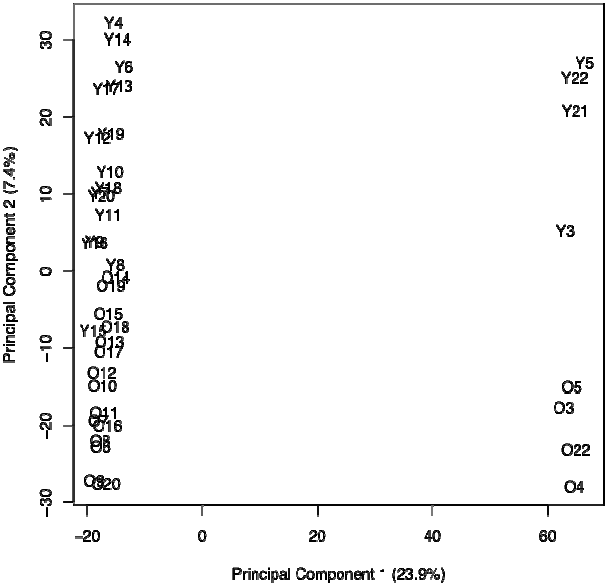

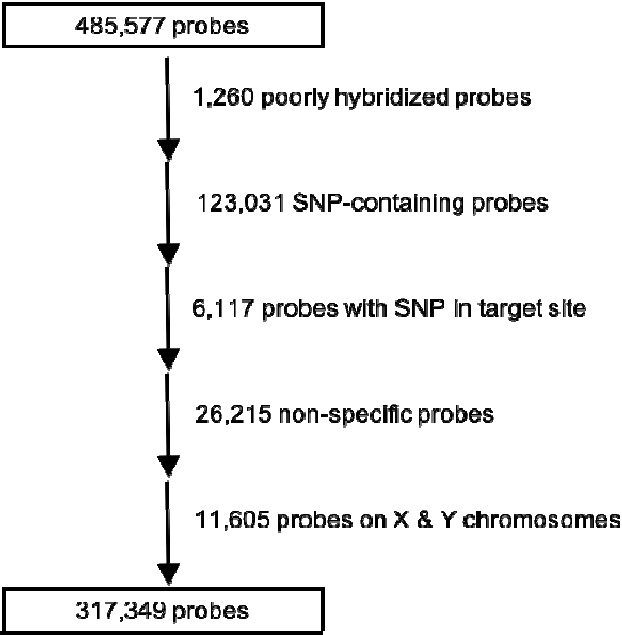
A B


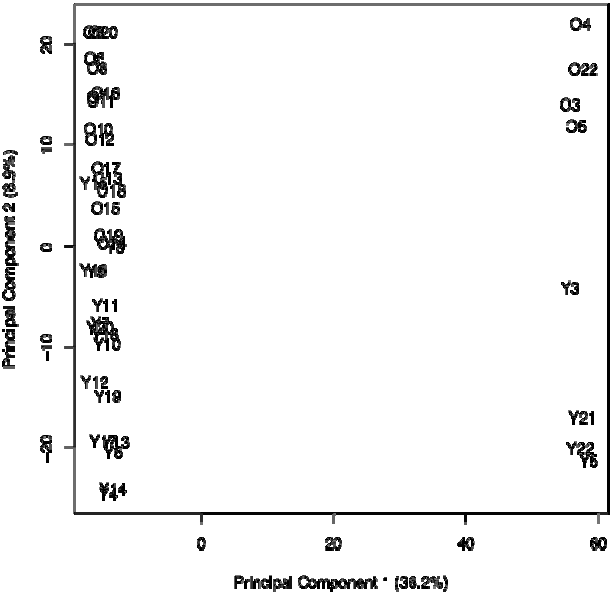
**C D**


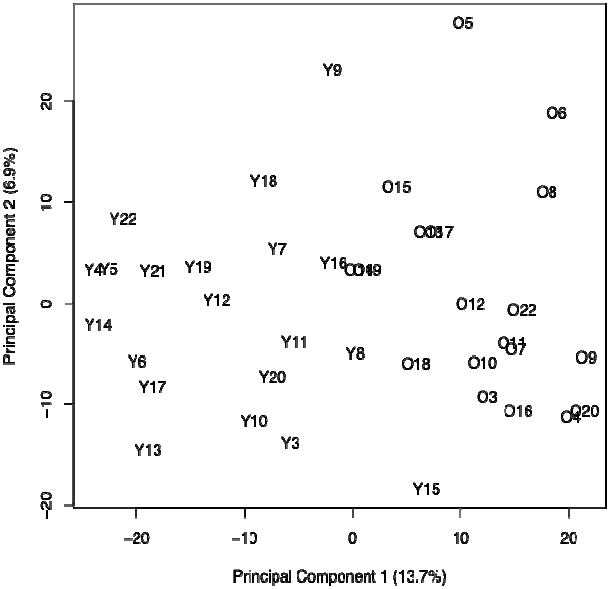


**Supplemental Figure 1**. **Subjects segregated by age group on MDS plot after probe filtering**. (A) Schematic representation of probe filtering. (B) MDS performed prior to any probe filtering (data from 485,577 probes) showed that subjects were distinctly segregated by sex.

(C) Segregation by sex remained after data from poorly hybridized probes, SNP-containing probes, probes with a SNP in the target site, and non-specific probes were filtered out. (D) After filtering out data from probes associated with X and Y chromosomes, samples were no longer segregated by sex but evident segregation based on age group emerged (data from 317,349 probes). Y = Younger Group, O = Older group.

**Supplemental Figure 2**

**2**

**Odds Ratio**

| ***** | | | | |
| --- | --- | --- | --- | --- |
| ***** | | |  |  |
|  |  |  |  |  |

**1**

**0**

**aDMRs Expression-Correlating aDMRs**

**Supplemental Figure 2. Expression-based MDD-associated genes are enriched in aDMRs and expression-correlating aDMRs**. Genes that were previously found to be differentially expressed in the postmortem brains of subjects with MDD are enriched in aDMRs and expression-correlating aDMRs.*, p < 0.05.

### Supplemental Figure 3


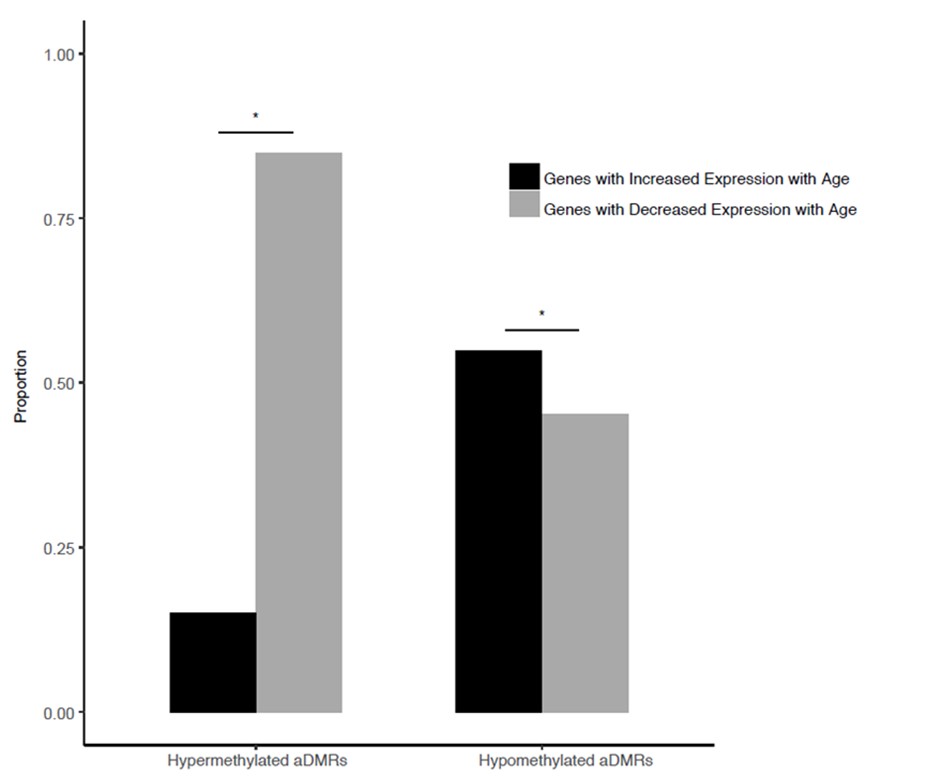


**Supplemental Figure 3. Direction of age-associated changes in DNA methylation and expression are inversely related for genes at which age-associated changes in DNA methylation and gene expression converge.** Decreased expression with age is 2.29 times more likely for those genes to which a hypermethylated aDMR is annotated (p=0.0002); and increased expression with age is 4.17 times more likely for those genes to which a hypomethylated aDMR is annotated (p=4.3*10-11). aDMR, age-associated differentially methylated regions, * indicates statistical significance.

0.2

0.3

**Supplemental Figure 4. Neuron-to-glia proportion does not differ between younger and older age groups.** Neuron-to-glia proportion in the sample from each subject was estimated using a model based on DNA methylation values from many cell epigenotype specific sites (21). The estimated neuron-to-glia proportion for the sample from each subject in the primary dataset is represented here. The younger and older group averages did not differ between (p=0.26). O, older, Y, younger.

**Neuron-to-Glia Proportion**

0.4

*O*3 O4 O5 O6 O7 O8 O9 O10 O11 O12 O13 O14 O15 O16 O17 O18 O19 O20 O22 Y3 Y4 Y5 Y6 Y7 Y8 Y9 Y10 Y11 Y12 Y13 Y14 Y15 Y16 Y17 Y18 Y19 Y20 Y21 Y22

0.0

0.1
